# Response: No need to match: a comment on Bach, Nicholson, and Hudson's “Affordance-Matching Hypothesis”

**DOI:** 10.3389/fnhum.2015.00685

**Published:** 2015-12-22

**Authors:** Patric Bach, Toby Nicholson, Matthew Hudson

**Affiliations:** School of Psychology, Cognition Institute, University of PlymouthPlymouth, UK

**Keywords:** affordances, action understanding, action prediction, object function, object manipulation

We are grateful for Uithol and Maranesi's (2014) insightful comments on our article “The affordance-matching hypothesis: How objects guide action understanding and prediction” (Bach et al., 2014). There, we argued that action understanding is not well-accounted for by process in which observed actions are simply matched, based on kinematic information, to an action in one's motor repertoire. Instead, we proposed that action understanding draws heavily on object information. Humans represent objects in terms of both (1) the goals that can be achieved with them (function knowledge), and (2) the specific motor behaviors required to achieve these goals (manipulation knowledge). This knowledge can make a major contribution to action observation, allowing observers not only to *infer* the goals someone wants to achieve with an object (via function knowledge) but also to *predict* the actions that this person would need to carry out to achieve these goals (via manipulation knowledge).

A key question in such a view is what derives the affordances—the known manipulations—of objects handled by other people. As Uithol and Maranesi rightly point out, and as we conclude in our article, canonical neurons are an unlikely candidate. While canonical neurons indeed seem to encode actions one can perform with an object (grasping, tearing), their firing is restricted to the peripersonal space, coding for actions the monkey could do *itself*. A much better candidate are the mirror-canonical neurons discovered by Bonini et al. (2014), which fulfill a similar role in the peripersonal space of other people. They fire both when the monkey sees an object, and when seeing someone else perform an appropriate action on the object. The Bonini et al. (2014) study was published shortly before our article, and we were only able to discuss it briefly in our paper. Yet, the response properties of these neurons match the predictions of affordance matching perfectly, and we are grateful to Uithol and Maranesi for further highlighting them. They nicely complement the wealth of behavioral evidence that reveal that observers extract object affordances for other people, even outside their own peripersonal space (for a review, see Creem-Regehr et al., 2013), and that mental simulation of hand-object interactions shows similarly lateralized motor activity as when actually performing such manipulations (e.g., when Borghi and Scorolli, 2009; Marino et al., 2012).

Next to highlighting this supportive evidence, Uithol and Maranesi provide two challenges for the affordance-matching hypothesis. First, we had argued that mirror neurons are not independent action recognizers. Instead, their purpose is confirmatory: they check whether one of the object's potential manipulations is indeed occurring (e.g., opening a peanut, grasping an apple; for similar arguments, see Kilner et al., 2007; Csibra, 2008). Support for this idea comes, among other findings reviewed in our article, from the observation that mirror neurons do not fire for a motor act in isolation, but only when it is directed to an appropriate object (Gallese et al., 1996) and that firing subsides quickly when the hand deviates from the predicted path. In contrast to this view, Uithol and Maranesi argue that mirror neurons could also recognize actions independently. They point to the audiovisual mirror neurons discovered by Kohler et al. (Kohler et al., 2002; Keysers et al., 2003). These neurons fire not only when an object-directed action is seen, but also when it is merely *heard* (e.g., the sound of a peanut breaking). As sound provides no object information, Uithol and Maranesi argue there is no prior affordance against which the action can be matched, arguing against an affordance matching interpretation of mirror neurons.

However, in our article, we specifically considered such cases in which action recognition occurs with little prior object information (e.g., because objects are hidden from view or actions are pantomimed). We argued that, in such cases, the action would not be matched to a seen object, but to a much greater variety of affordances of objects in memory. Identifying such a match would therefore be slow and effortful, unless the observed movements are highly idiosyncratic, or the potential objects had already been constrained by the prior context. Strikingly, all these considerations seem to apply to the original Kohler studies. They tested a very limited set of six actions, on which the monkeys were extensively trained, and which were shown repeatedly, in random order, during the experiment. Thus, while vision did not provide object information directly, the potential set of objects was nevertheless highly constrained, and the heard actions could be efficiently matched to one of these alternatives. To our knowledge this has not been tested yet in monkeys, but affordance matching would predict that these auditory mirror neuron responses would be very much delayed or reduced, if no such prior experimental object context would be available.

Finally, we had proposed that function and manipulation knowledge about objects could interact, in a productive manner, during action observation. Knowing somebody's goals will predict exactly which manipulations are required with an object to achieve this goal, supporting action *prediction*. Conversely, recognizing a known way of manipulating an object allows one to infer which of the object's functions the actor wants to realize, supporting action *interpretation*. Uithol and Maranesi argue that a single process, similar to pattern completion processes in vision, could account for both. In this view, an object representation linking a goal (driving in a nail), an object (a hammer), and a required manipulation (forceful downwards movements) provides such a pattern, which is filled in if one aspect is missing (as long as the overall pattern is recognized). We are not averse to this possibility. The affordance-matching hypothesis is relatively agnostic as to how the proposed mechanism is implemented. What we would like to argue—and this was the purpose of the paper—is that as soon as an architecture linking objects, goals and body movements is established it can be used for both purposes: prediction (when likely movements are inferred from objects and goals) and interpretation (when likely goals are inferred from how the object is manipulated). Thus, rather than reflecting different processes, prediction and understanding are different *processing outcomes* that arise (a) from the completeness of the stimulus, and (b) from the task. For example, coordinating our own action with that of another person (e.g., handing over an object) requires efficient prediction. In contrast, longer-term predictions about others' behavior require knowledge of their goals.

Importantly, though, and this is perhaps the main point of disagreement, we do not believe that, even if there was such a pattern completion process, this would negate the “need to match.” In vision, only the simplest possible patterns can be “filled in” without recourse to prior knowledge, for example, in cases of edge extensions or extrapolation of retinal motion. Instead, it requires that a matching pattern at a higher cortical level is activated (Rao and Ballard, 1999). Completion is possible precisely because this matching representation can provide the missing information. This is not an out-dated “cognitivist” assumption either: Recent predictive coding models see it as the core of general brain function, across all levels of the cortical hierarchy (Barsalou, 2009; Friston and Kiebel, 2009). The brain constantly forms higher-level hypotheses about the environment, which are propagated downwards and tested against the sensory input. Prediction errors are fed back upwards so that matching hypothesis can be confirmed, and mismatching ones are revised until they match the sensory input. The affordance matching view is directly informed by these views. Objects provide both hypotheses about potential goals (the object's function), and a means for testing them against the currently observed action (the associated manipulations). Object knowledge, therefore, provides the “patterns” against which seen actions can be compared, and from which their goal can be derived.

We would like to end by noting that in the year since publication several studies have provided evidence for the different components of our model. For example, Thioux and Keysers (2015) demonstrated direct links between connectivity in parietal-premotor “mirror” circuits and the ability to anticipate which of two objects someone else is going to grasp, providing evidence for an encoding of object–action affordance relationships in these areas. Similarly, Schubotz et al. (2014) showed that activity in some of these regions increased parametrically with the number of actions afforded by the goal object, in line with the notion that observed actions are indeed matched against action “hypotheses” derived from objects. Finally, Maranesi et al. (2014) revealed predictive firing of mirror neurons before action initiation, if the context (a go signal) implied that the observed actor had a goal of reaching toward the object. This provided direct support for the idea that prior goal assumptions specify which action someone will carry out with an object, which can then be tested against the actual visual input. Indeed, we have recently provided direct evidence for this idea, by showing that top-down predictions directly feed into even low-level perceptual representations of observed motor acts, biasing them further toward the assumed goals than they really were (Hudson et al., 2015, 2016).

## Conflict of interest statement

The authors declare that the research was conducted in the absence of any commercial or financial relationships that could be construed as a potential conflict of interest.
